# Surgical site infection in elderly patients

**DOI:** 10.1186/1471-2318-11-S1-A37

**Published:** 2011-08-24

**Authors:** M Minutolo, G Blandino, R Lanteri, S Puleo, V Minutolo

**Affiliations:** 1 Department of Microbiological Sciences, Organ Transplantation and Advanced Technologies University of Catania, Italy; 2 Department of Surgical Sciences, Organ Transplantation and Advanced Technologies University of Catania, Italy

## Background

The surgical site infection (SSI) is an unforeseen event that complicates patient post-operative care with a negative impact on results and may cause further surgery which could lead to the patient’s death [1]**.** Furthermore, the bacteriological analysis of the surgical wound could be a predictive method of the surgical site infection after elective abdominal surgery [2,3]. Moreover post-operative infections lead to a growth of hospital costs, home medical care, social and psychological. Starting from this background it is clear how SSI control has a professional, ethical and economic value. Antibiotic prophylaxis is useful in preventing wound infection and limiting the negative effects of infection on the patient. The aim of this study is to evaluate the role of age and antibiotic prophylaxis in the incidence of surgical wound infection in patients undergoing elective abdominal surgery. Microbiological analysis of wound, surgical treatment, hospital and anesthesiological risk were evaluated.

## Patients and methods

**Figure 1:**
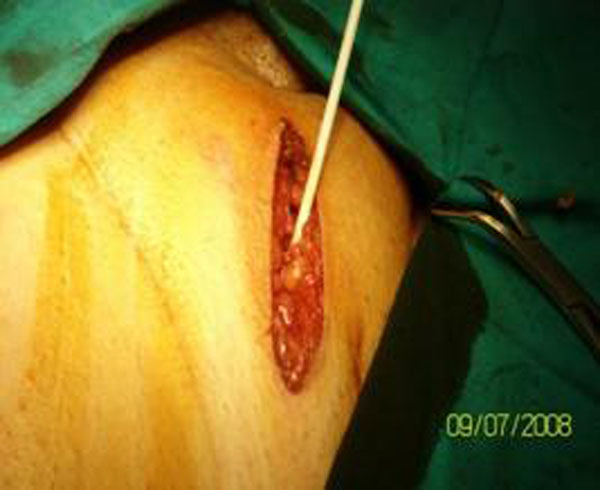
Subcutaneous tissue wound

78 patients, divided in group A (21pt age >70 years) and group B (57pt age < 70 years), were studied to assess the risk factors of abdominal surgical wound infection. Subcutaneous tissue swabs made after closure of the fascia and before suturing the skin were examined (Fig. 1). The indications for surgery were benign and malignant diseases such as abdominal hernias, gallstones, colorectal cancer, gastric cancer, kidney cancer, acute perforation of duodenal ulcer and choking hernia. All surgical interventions were classified as clean or clean / contaminated. Antibiotic prophylaxis was performed only in 48 patients 60 minutes before surgery. The presence of wound infection was documented during the hospital stay and for up to 4 weeks after surgery.

## Results

Of the 78 swabs, 30 were considered sterile. Of the 48 positive swabs microbiological analysis (47 isolations monomicrobici and only one polymicrobial) only 4 were associated with a wound infection (two strains of Escherichia coli, one Enterobacter cloacae and one Candida albicans). Moreover, in two speeches, in which the swab of the wound showed no microbial growth, there was an infection caused by Bacteroides fragilis (table 1). The incidence of surgical wound infection in group A (> 70 years) was of 9.52%, while in group B (<70 years) of 8.77% and 4.25% overall in patients undergoing antibiotic prophylaxis and 12.90% of patients who had not performed antibiotic prophylaxis (Tables 2-3).

**Table 1:** **Our series.** Surgical site infections. Our series 7/78 (8.97%)

**Isolated species** ( patient )	**Surgical mean time** (min.)
*Escherichia coli* ( *group A* )	**120**
*Escherichia coli* ( *group B* )	**130**
*Enterobacter cloacae* ( *group B* )	**75**
*Bacteroides fragilis* ( *group B* )	**140**
*Bacteroides fragilis* ( *group B* )	**110**
*Candida albicans* ( *grouo B* )	**190**
*Escherichia coli* ( *group A* )	**200**

**Table 2:** Relationship between surgical site infection, patient’s age, surgical time and anesthesiological risk

Patients	Surgical site contamination (%)	Surgical site infection ( % )	Surgical mean time (min)	ASA SCORE
Group A	11/21 (52.38%)	2/21 (9,52%)	160	III-IV
Group B	37/57 ( 64.91%)	5/57 (8,77%)	129	III

**Table 3:** Relationship between surgical site contamination and sex

Sex	Surgical site contamination (%)
M	37/51 ( 72.54 % )
F	11/27 (40.74% )

## Conclusions

In our series no statistical differences were observed between the two groups in relation to the age.
